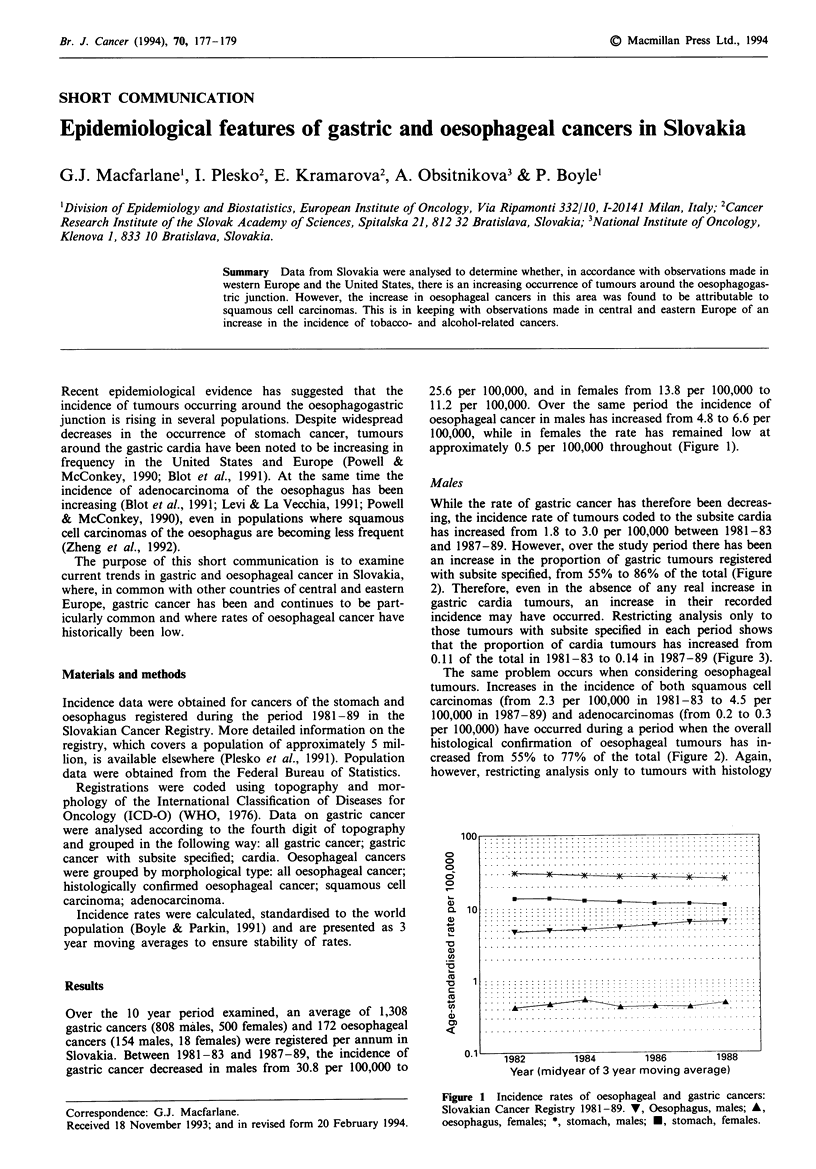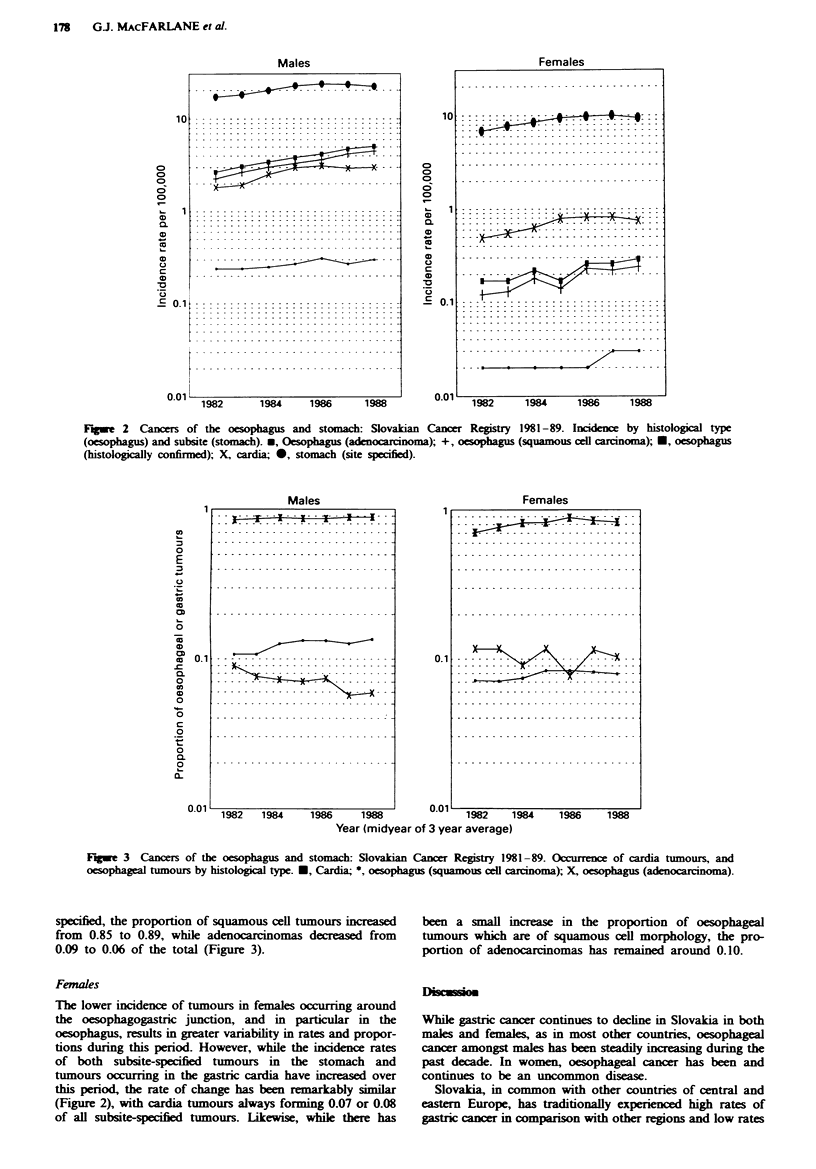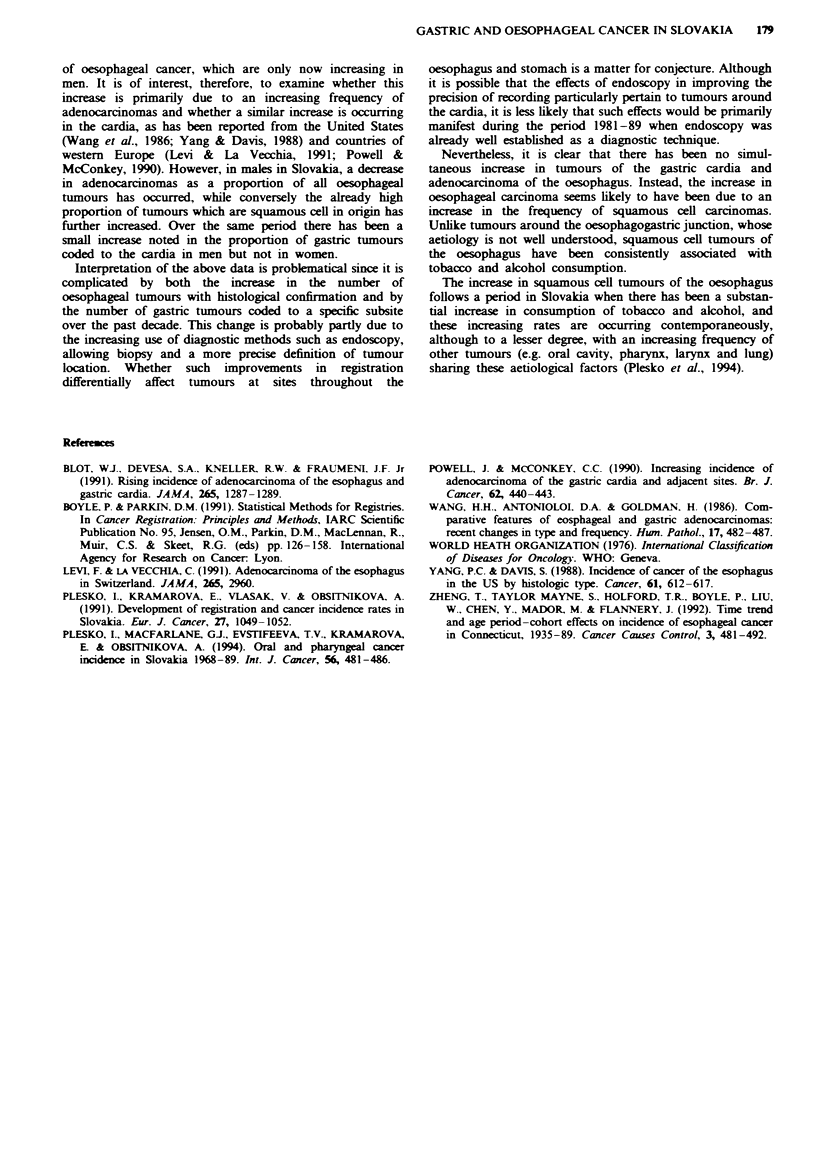# Epidemiological features of gastric and oesophageal cancers in Slovakia.

**DOI:** 10.1038/bjc.1994.272

**Published:** 1994-07

**Authors:** G. J. Macfarlane, I. Plesko, E. Kramarova, A. Obsitnikova, P. Boyle

**Affiliations:** Division of Epidemiology and Biostatistics, European Institute of Oncology, Milan, Italy.

## Abstract

Data from Slovakia were analysed to determine whether, in accordance with observations made in western Europe and the United States, there is an increasing occurrence of tumours around the oesophagogastric junction. However, the increase in oesophageal cancers in this area was found to be attributable to squamous cell carcinomas. This is in keeping with observations made in central and eastern Europe of an increase in the incidence of tobacco- and alcohol-related cancers.


Br. J. Cancer (1994), 70, 177–179 © Macmillan Press Ltd., 1994

SHORT COMMUNICATION

### Epidemiological features of gastric and oesophageal cancers in Slovakia

G.J. Macfarlane[1], I. Plesko[2], E. Kramarova[2], A. Obsitnikova[3] & P. Boyle[1]

[1]*Division of Epidemiology and Biostatistics, European Institute of Oncology, Via Ripamonti 332/10, I-20141 Milan, Italy;* [2]*Cancer Research Institute of the Slovak Academy of Sciences, Spitalska 21, 812 32 Bratislava, Slovakia;* [3]*National Institute of Oncology, Klenova 1, 833 10 Bratislava, Slovakia.*

**Summary** Data from Slovakia were analysed to determine whether, in accordance with observations made in western Europe and the United States, there is an increasing occurrence of tumours around the oesophagogastric junction. However, the increase in oesophageal cancers in this area was found to be attributable to squamous cell carcinomas. This is in keeping with observations made in central and eastern Europe of an increase in the incidence of tobacco- and alcohol-related cancers.

Recent epidemiological evidence has suggested that the incidence of tumours occurring around the oesophagogastric junction is rising in several populations. Despite widespread decreases in the occurrence of stomach cancer, tumours around the gastric cardia have been noted to be increasing in frequency in the United States and Europe (Powell & McConkey, 1990; Blot *et al.*, 1991). At the same time the incidence of adenocarcinoma of the oesophagus has been increasing (Blot *et al.*, 1991; Levi & La Vecchia, 1991; Powell & McConkey, 1990), even in populations where squamous cell carcinomas of the oesophagus are becoming less frequent (Zheng *et al.*, 1992).

The purpose of this short communication is to examine current trends in gastric and oesophageal cancer in Slovakia, where, in common with other countries of central and eastern Europe, gastric cancer has been and continues to be particularly common and where rates of oesophageal cancer have historically been low.

### Materials and methods

Incidence data were obtained for cancers of the stomach and oesophagus registered during the period 1981–89 in the Slovakian Cancer Registry. More detailed information on the registry, which covers a population of approximately 5 million, is available elsewhere (Plesko *et al.*, 1991). Population data were obtained from the Federal Bureau of Statistics.

Registrations were coded using topography and morphology of the International Classification of Diseases for Oncology (ICD-O) (WHO, 1976). Data on gastric cancer were analysed according to the fourth digit of topography and grouped in the following way: all gastric cancer; gastric cancer with subsite specified; cardia. Oesophageal cancers were grouped by morphological type: all oesophageal cancer; histologically confirmed oesophageal cancer; squamous cell carcinoma; adenocarcinoma.

Incidence rates were calculated, standardised to the world population (Boyle & Parkin, 1991) and are presented as 3 year moving averages to ensure stability of rates.

### Results

Over the 10 year period examined, an average of 1,308 gastric cancers (808 males, 500 females) and 172 oesophageal cancers (154 males, 18 females) were registered per annum in Slovakia. Between 1981–83 and 1987–89, the incidence of gastric cancer decreased in males from 30.8 per 100,000 to 25.6 per 100,000, and in females from 13.8 per 100,000 to 11.2 per 100,000. Over the same period the incidence of oesophageal cancer in males has increased from 4.8 to 6.6 per 100,000, while in females the rate has remained low at approximately 0.5 per 100,000 throughout (Figure 1).

### *Males*

While the rate of gastric cancer has therefore been decreasing, the incidence rate of tumours coded to the subsite cardia has increased from 1.8 to 3.0 per 100,000 between 1981–83 and 1987–89. However, over the study period there has been an increase in the proportion of gastric tumours registered with subsite specified, from 55% to 86% of the total (Figure 2). Therefore, even in the absence of any real increase in gastric cardia tumours, an increase in their recorded incidence may have occurred. Restricting analysis only to those tumours with subsite specified in each period shows that the proportion of cardia tumours has increased from 0.11 of the total in 1981–83 to 0.14 in 1987–89 (Figure 3).

The same problem occurs when considering oesophageal tumours. Increases in the incidence of both squamous cell carcinomas (from 2.3 per 100,000 in 1981–83 to 4.5 per 100,000 in 1987–89) and adenocarcinomas (from 0.2 to 0.3 per 100,000) have occurred during a period when the overall histological confirmation of oesophageal tumours has increased from 55% to 77% of the total (Figure 2). Again, however, restricting analysis only to tumours with histology

**Figure 1** Incidence rates of oesophageal and gastric cancers: Slovakian Cancer Registry 1981–89. ▼, Oesophagus, males; ▲, oesophagus, females; *, stomach, males; ■, stomach, females.

Correspondence: G.J. Macfarlane.
Received 18 November 1993; and in revised form 20 February 1994.

Figure 2 Cancers of the oesophagus and stomach: Slovakian Cancer Registry 1981–89. Incidence by histological type (oesophagus) and subsite (stomach). ■, Oesophagus (adenocarcinoma); +, oesophagus (squamous cell carcinoma); ■, oesophagus (histologically confirmed); X, cardia; ●, stomach (site specified).

Figure 3 Cancers of the oesophagus and stomach: Slovakian Cancer Registry 1981–89. Occurrence of cardia tumours, and oesophageal tumours by histological type. ■, Cardia; *, oesophagus (squamous cell carcinoma); X, oesophagus (adenocarcinoma).

specified, the proportion of squamous cell tumours increased from 0.85 to 0.89, while adenocarcinomas decreased from 0.09 to 0.06 of the total (Figure 3).

*Females*

The lower incidence of tumours in females occurring around the oesophagogastric junction, and in particular in the oesophagus, results in greater variability in rates and proportions during this period. However, while the incidence rates of both subsite-specified tumours in the stomach and tumours occurring in the gastric cardia have increased over this period, the rate of change has been remarkably similar (Figure 2), with cardia tumours always forming 0.07 or 0.08 of all subsite-specified tumours. Likewise, while there has been a small increase in the proportion of oesophageal tumours which are of squamous cell morphology, the proportion of adenocarcinomas has remained around 0.10.

### Discussion

While gastric cancer continues to decline in Slovakia in both males and females, as in most other countries, oesophageal cancer amongst males has been steadily increasing during the past decade. In women, oesophageal cancer has been and continues to be an uncommon disease.

Slovakia, in common with other countries of central and eastern Europe, has traditionally experienced high rates of gastric cancer in comparison with other regions and low rates of oesophageal cancer, which are only now increasing in men. It is of interest, therefore, to examine whether this increase is primarily due to an increasing frequency of adenocarcinomas and whether a similar increase is occurring in the cardia, as has been reported from the United States (Wang *et al.*, 1986; Yang & Davis, 1988) and countries of western Europe (Levi & La Vecchia, 1991; Powell & McConkey, 1990). However, in males in Slovakia, a decrease in adenocarcinomas as a proportion of all oesophageal tumours has occurred, while conversely the already high proportion of tumours which are squamous cell in origin has further increased. Over the same period there has been a small increase noted in the proportion of gastric tumours coded to the cardia in men but not in women.

Interpretation of the above data is problematical since it is complicated by both the increase in the number of oesophageal tumours with histological confirmation and by the number of gastric tumours coded to a specific subsite over the past decade. This change is probably partly due to the increasing use of diagnostic methods such as endoscopy, allowing biopsy and a more precise definition of tumour location. Whether such improvements in registration differentially affect tumours at sites throughout the oesophagus and stomach is a matter for conjecture. Although it is possible that the effects of endoscopy in improving the precision of recording particularly pertain to tumours around the cardia, it is less likely that such effects would be primarily manifest during the period 1981–89 when endoscopy was already well established as a diagnostic technique.

Nevertheless, it is clear that there has been no simultaneous increase in tumours of the gastric cardia and adenocarcinoma of the oesophagus. Instead, the increase in oesophageal carcinoma seems likely to have been due to an increase in the frequency of squamous cell carcinomas. Unlike tumours around the oesophagogastric junction, whose aetiology is not well understood, squamous cell tumours of the oesophagus have been consistently associated with tobacco and alcohol consumption.

The increase in squamous cell tumours of the oesophagus follows a period in Slovakia when there has been a substantial increase in consumption of tobacco and alcohol, and these increasing rates are occurring contemporaneously, although to a lesser degree, with an increasing frequency of other tumours (e.g. oral cavity, pharynx, larynx and lung) sharing these aetiological factors (Plesko *et al.*, 1994).

### References

BLOT, W.J., DEVESA, S.A., KNELLER, R.W. & FRAUMENI, J.F. Jr (1991). Rising incidence of adenocarcinoma of the esophagus and gastric cardia. *JAMA*, **265**, 1287–1289.

BOYLE, P. & PARKIN, D.M. (1991). Statistical Methods for Registries. In *Cancer Registration: Principles and Methods*, IARC Scientific Publication No. 95, Jensen, O.M., Parkin, D.M., MacLennan, R., Muir, C.S. & Skeet, R.G. (eds) pp. 126–158. International Agency for Research on Cancer: Lyon.

LEVI, F. & LA VECCHIA, C. (1991). Adenocarcinoma of the esophagus in Switzerland. *JAMA*, **265**, 2960.

PLESKO, I., KRAMAROVA, E., VLASAK, V. & OBSITNIKOVA, A. (1991). Development of registration and cancer incidence rates in Slovakia. *Eur. J. Cancer*, **27**, 1049–1052.

PLESKO, I., MACFARLANE, G.J., EVSTIFEEVA, T.V., KRAMAROVA, E. & OBSITNIKOVA, A. (1994). Oral and pharyngeal cancer incidence in Slovakia 1968–89. *Int. J. Cancer*, **56**, 481–486.

POWELL, J. & McCONKEY, C.C. (1990). Increasing incidence of adenocarcinoma of the gastric cardia and adjacent sites. *Br. J. Cancer*, **62**, 440–443.

WANG, H.H., ANTONIOLOI, D.A. & GOLDMAN, H. (1986). Comparative features of esophageal and gastric adenocarcinomas: recent changes in type and frequency. *Hum. Pathol.*, **17**, 482–487.

WORLD HEATH ORGANIZATION (1976). *International Classification of Diseases for Oncology*. WHO: Geneva.

YANG, P.C. & DAVIS, S. (1988). Incidence of cancer of the esophagus in the US by histologic type. *Cancer*, **61**, 612–617.

ZHENG, T., TAYLOR MAYNE, S., HOLFORD, T.R., BOYLE, P., LIU, W., CHEN, Y., MADOR, M. & FLANNERY, J. (1992). Time trend and age period–cohort effects on incidence of esophageal cancer in Connecticut, 1935–89. *Cancer Causes Control*, **3**, 481–492.